# Supplementary figures and images for: Membrane-associated σ factors disrupt rRNA operon clustering in Escherichia coli
Source: PLoS Biol. 2025 Apr 17;23(4):e3003113. doi: 10.1371/journal.pbio.3003113 (PMC12037070; doi:10.1371/journal.pbio.3003113)

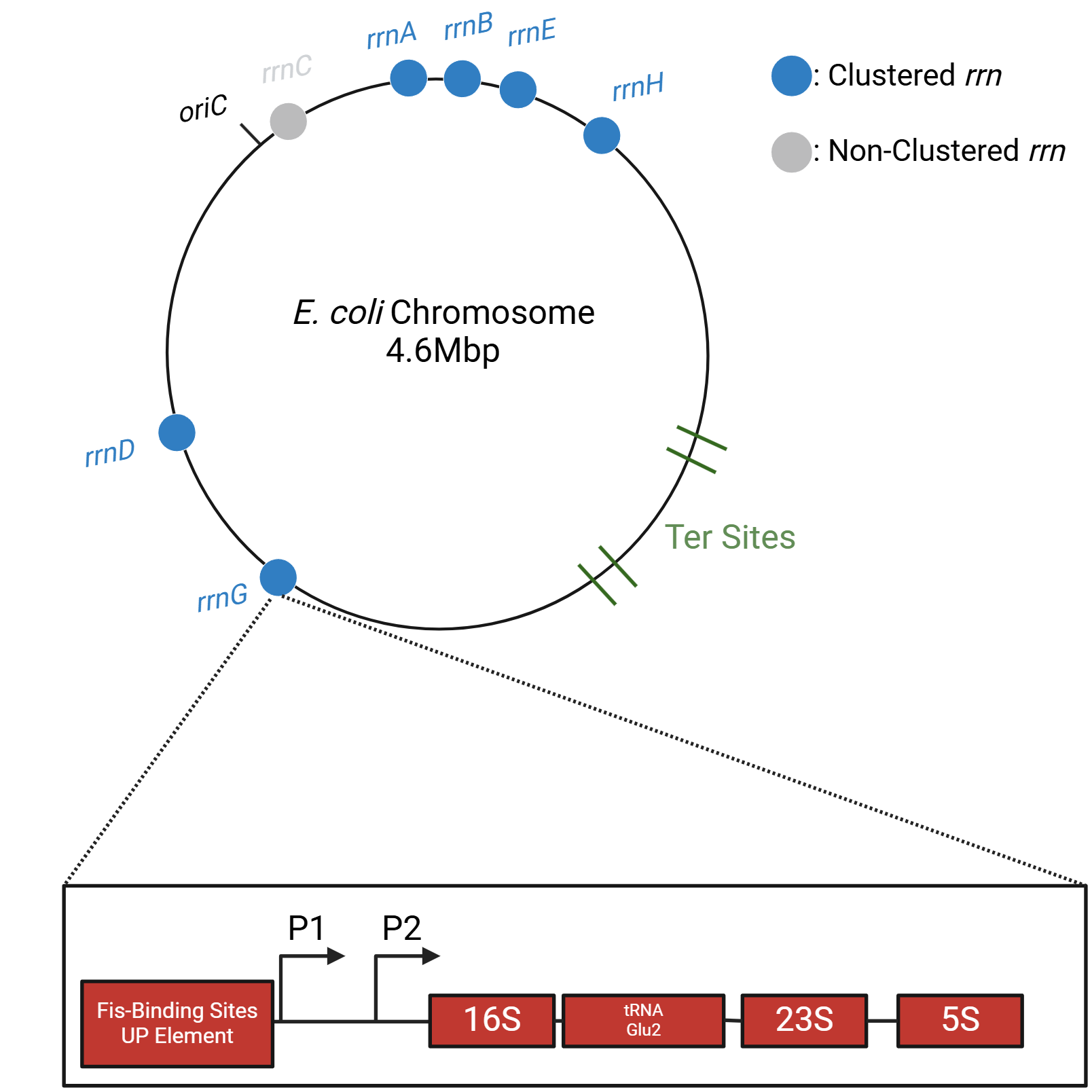

Supplement: S1 Fig — On the circular E. coli chromosome, replication originates at oriC, with two bidirectional replication forks traversing each arm (replicore), terminating within the Ter region. Locations of the rrn operons shown to cluster by two different methodologies (see main text) are indicated by blue-filled circles. Positions of the operons on the circular chromosome are not drawn to scale. Organization of a typical rrn operon is shown for rrnG, with tRNAGlu2 between 16S and 23S; some rrn loci possess distal tRNAs. P1 and P2 promoters are indicated by forward arrows. Created in BioRender. Ho, K. (2025) https://BioRender.com/g85e152. (TIFF) [file pbio.3003113.s001.tiff]

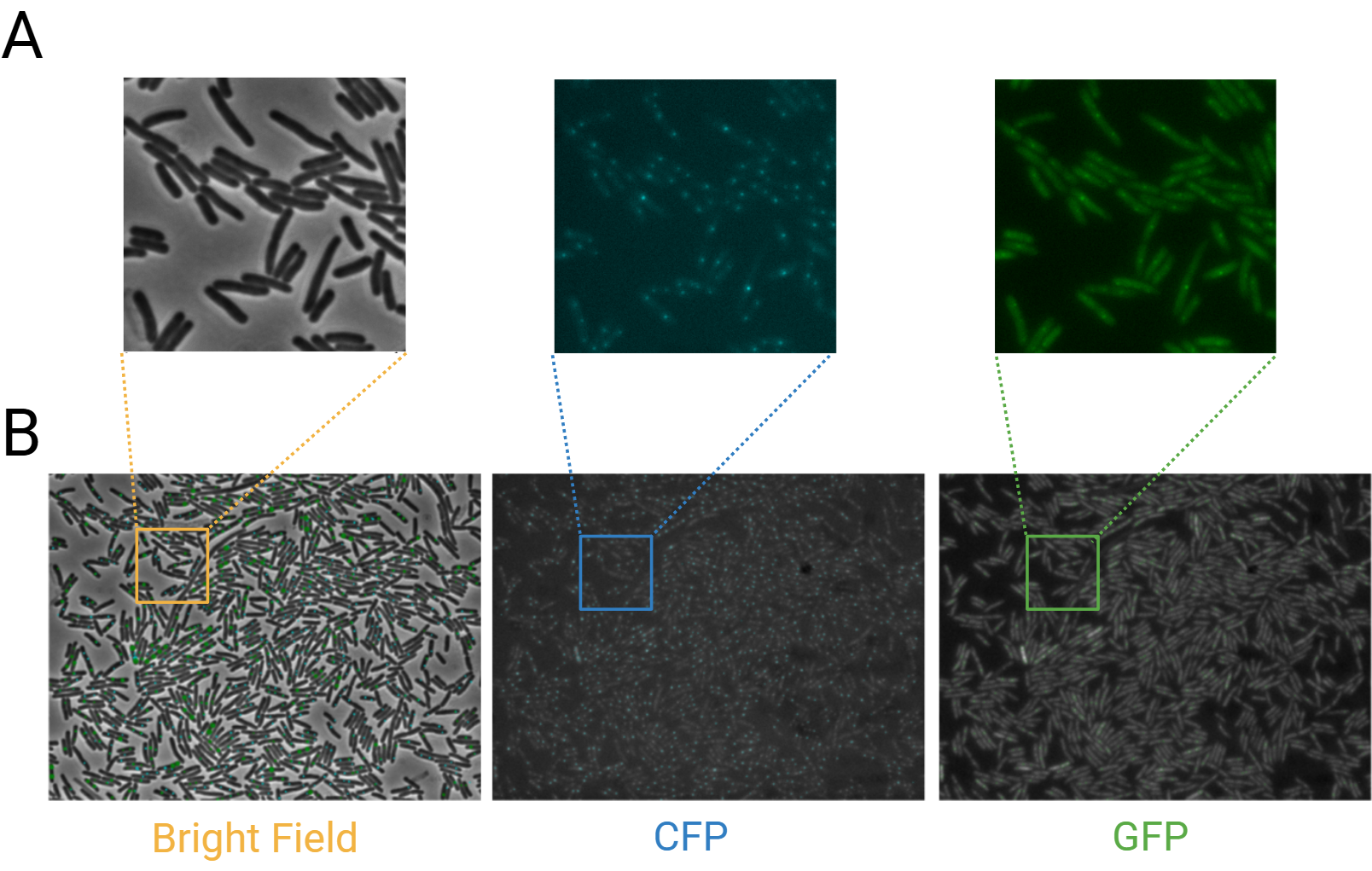

Supplement: S2 Fig — (A) Insets of a complete field of cells (phase contrast and fluorescence) shown in B. rrnA and rrnD were tagged with GFP-ParB and CFP-ParB, respectively, as described in Fig 1A, and processed as described in Fig 1B. (B) Complete field and position of identified foci. Created in BioRender. Ho, K. (2025) https://BioRender.com/p20w223. (TIFF) [file pbio.3003113.s002.tiff]

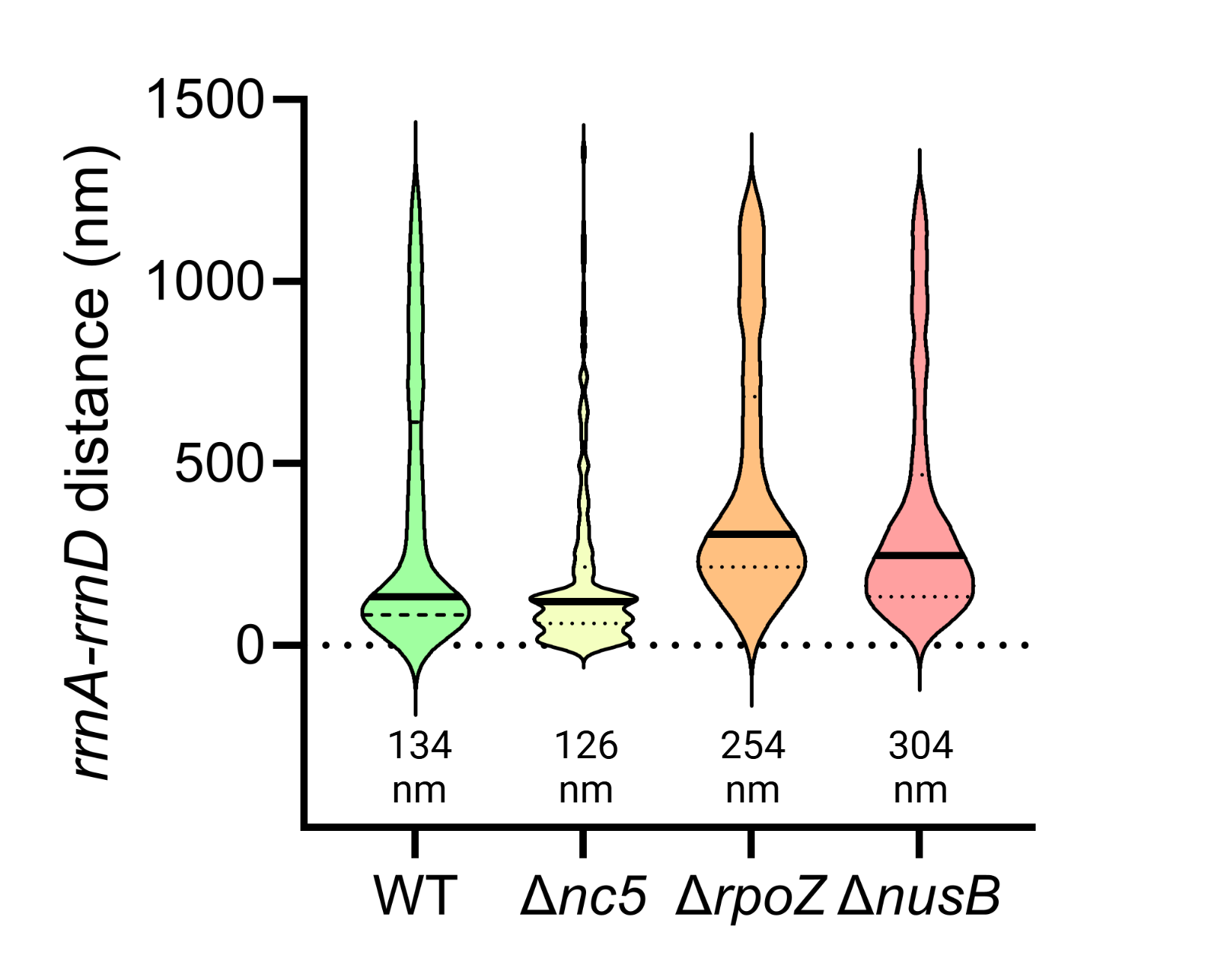

Supplement: S3 Fig — Effect of deletions of factors known to participate in RNAP condensate on rrnA-rrnD distance. Experimental conditions are similar to those in Fig 1. Data used for plotting S3 Fig graphs can be found in the Supplemental “S1 Data” file under appropriate figure headings. Created in BioRender. Ho, K. (2025) https://BioRender.com/a48g408. (TIFF) [file pbio.3003113.s003.tiff]

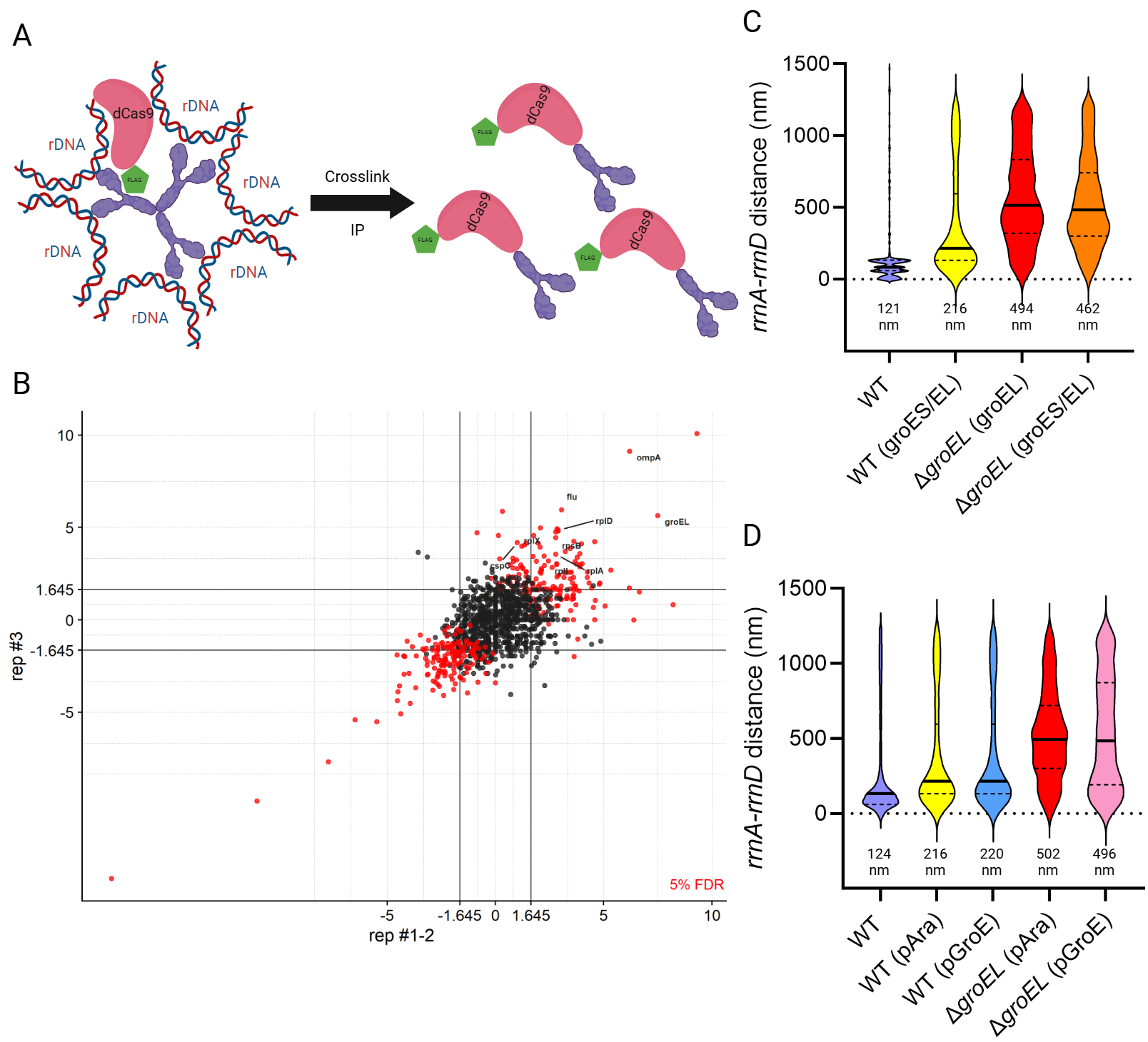

Supplement: S4 Fig — (A) Scheme for pulldown of proteins in the vicinity of all rrn loci. dCas9-FLAG (pink/green) was directed upstream of all 7 rrn loci by expressing sgRNA specific for each target. Formaldehyde was used to crosslink dCas9 to putative bridging factor(s) (purple). The dCas9-linked ‘complex’ was then immunoprecipitated and subjected to mass spectrometry. (B) Significantly enriched proteins in pulldown with gRNA targeting yeiP (rrnC). Each protein is identified by a circle. Each axis represents the z-score of each protein in separate experiments. Lines from the axes indicate the cut-off for enrichment. Red circles and black circles indicate proteins that fall above and below the False Discovery Rate (5%), respectively. Proteins significantly enriched (z-score>2.5) are in the top-right square (red balls with corresponding protein name). See S6 Fig for data obtained for the remaining gRNAs. (C) Distance between rrnA-D operons in WT and ∆groEL strains expressing either both groES/groEL or groEL alone from pAraBAD plasmid. Other descriptions as in Fig 1B. (D) Distance between rrnA-D in WT and ∆groEL strains expressing groES/groEL from either pAra or the native groE promoter. Data used for plotting S4 Fig graphs can be found in Supplemental “S1 Data” file under appropriate figure headings. Created in BioRender. Ho, K. (2025) https://BioRender.com/z72h116. (TIFF) [file pbio.3003113.s004.tiff]

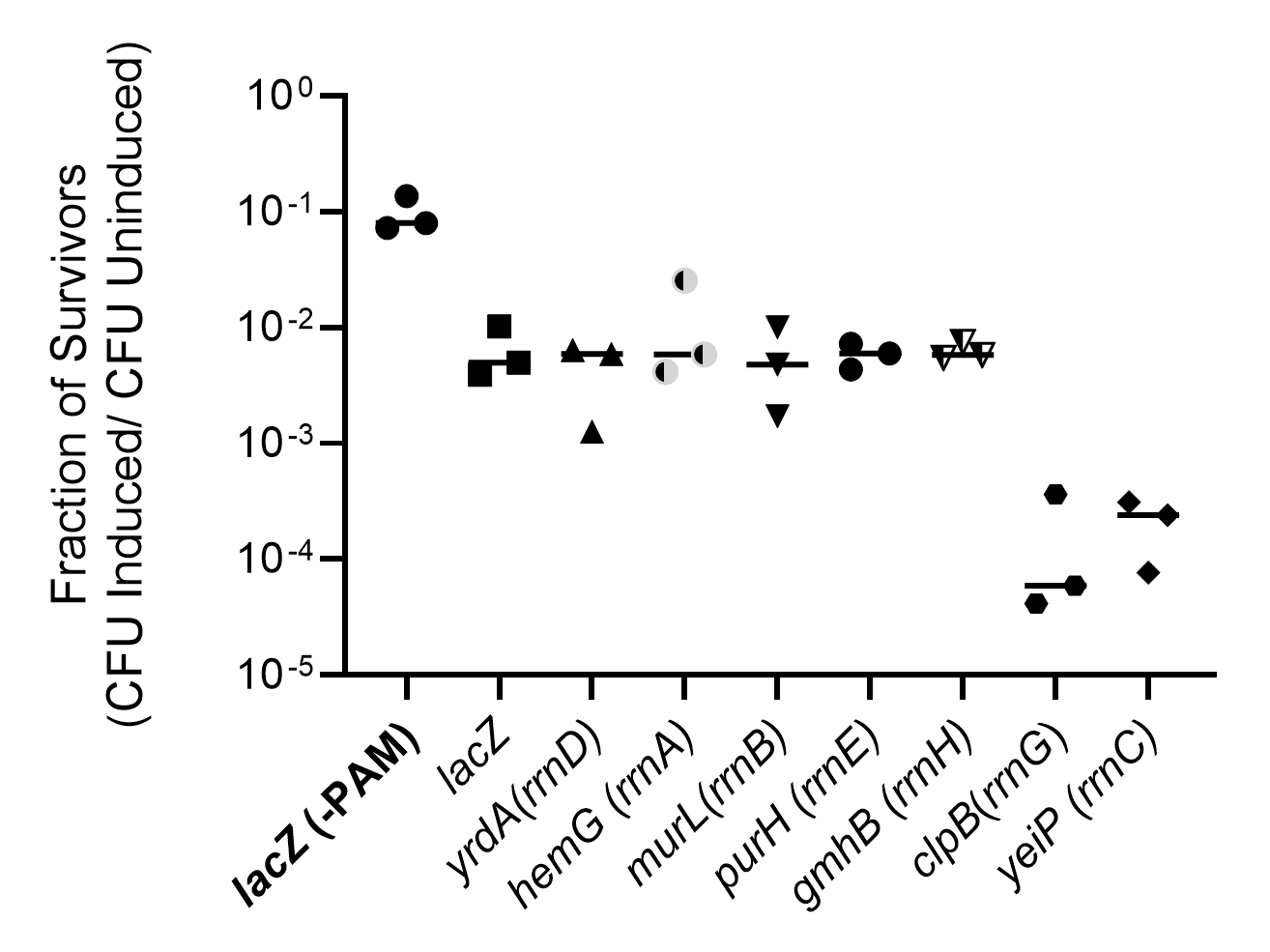

Supplement: S5 Fig — Lethality was determined by the fraction of CFU that survive induction of Cas9 and gRNA compared to the CFU of non-induced control (y-axis). Gene names on the x-axis indicate the targeted gene immediately upstream of the rrn operon indicated in parentheses. gRNA targeting lacZ without (bolded) and with the protospacer adjacent motif, were used as negative and positive controls, respectively. Data used for plotting S5 Fig graphs can be found in Supplemental “S1 Data” file under appropriate figure headings. (TIFF) [file pbio.3003113.s005.tiff]

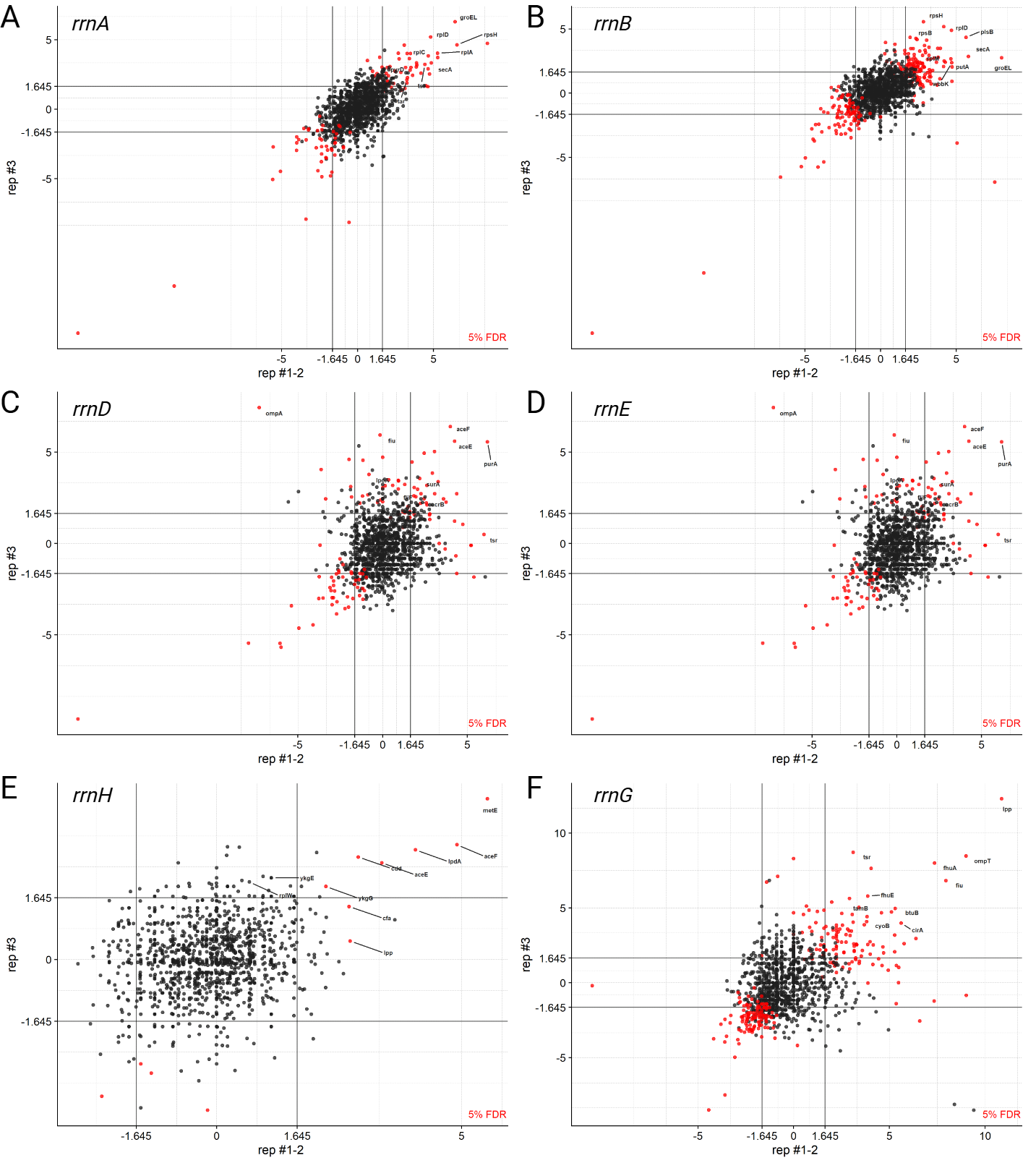

Supplement: S6 Fig — (A–F) Results from gRNAs directed to indicated rrn loci. See S4A Fig legend for other experimental details. Data used for plotting S6 Fig graphs for each gRNA pulldown can be found in S3–S9 Tables. Created in BioRender. Ho, K. (2025) https://BioRender.com/i51m760. (TIFF) [file pbio.3003113.s006.tiff]

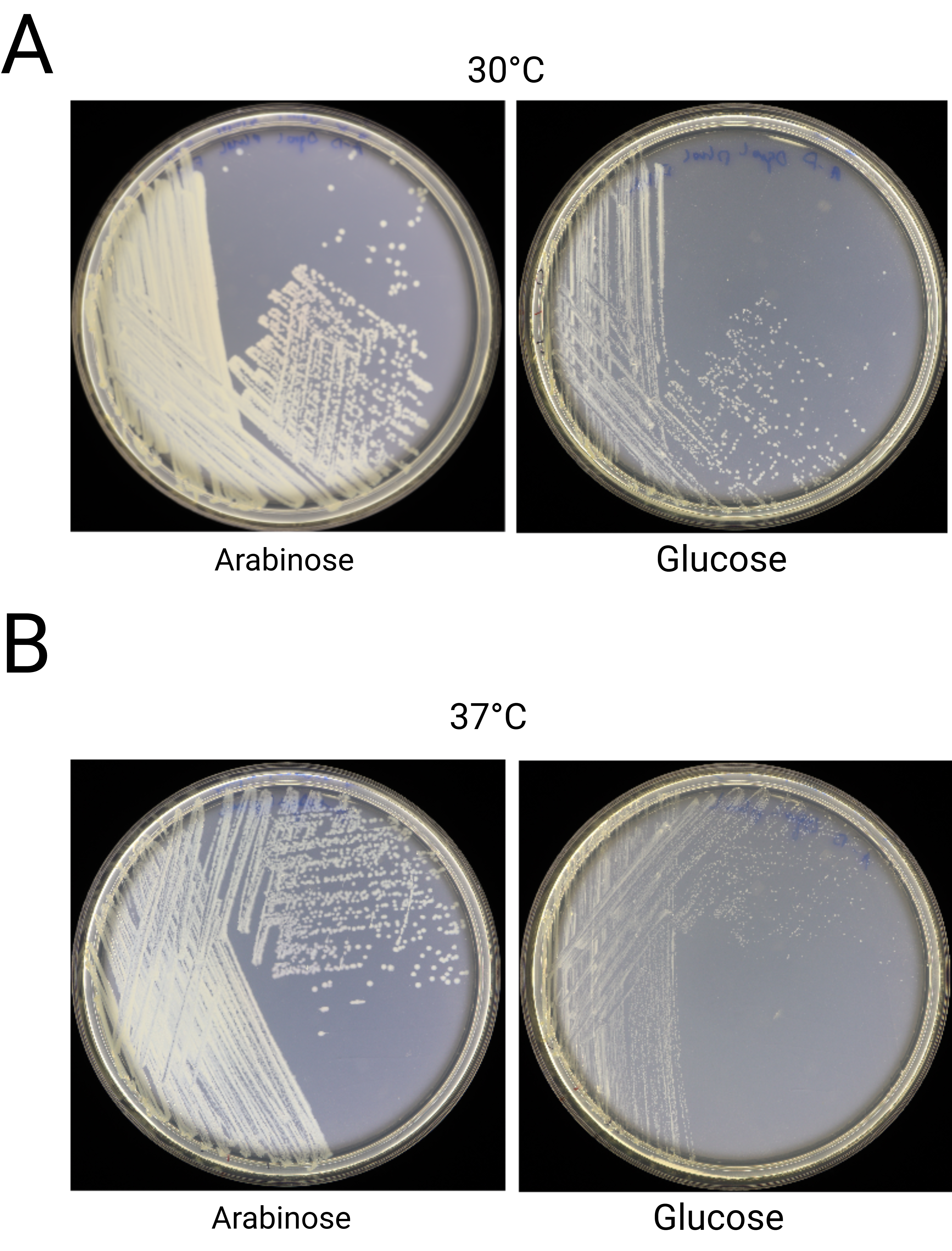

Supplement: S8 Fig — Colony morphologies upon induction and repression of GroEL with added arabinose or glucose, respectively, at 30 °C (A) and 37 °C (B). See S4D Fig and related text. Created in BioRender. Ho, K. (2025) https://BioRender.com/t72u237. (TIFF) [file pbio.3003113.s008.tiff]

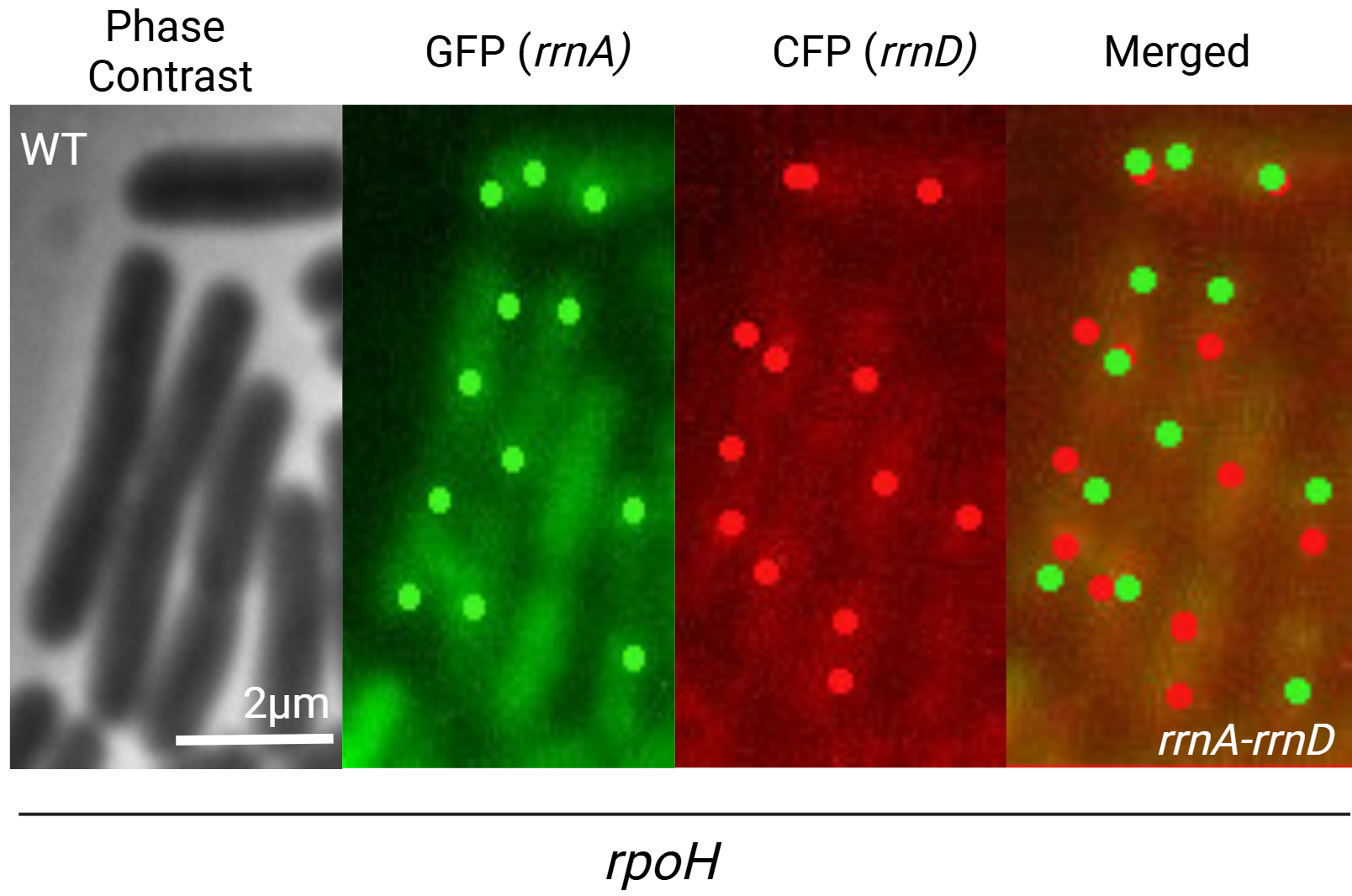

Supplement: S9 Fig — Representative image of rrnA-rrnD foci showing no loss of rrnA-GFP foci as seen with heat stress in Fig 1D. Created in BioRender. Ho, K. (2025) https://BioRender.com/t54z191. (TIFF) [file pbio.3003113.s009.tiff]

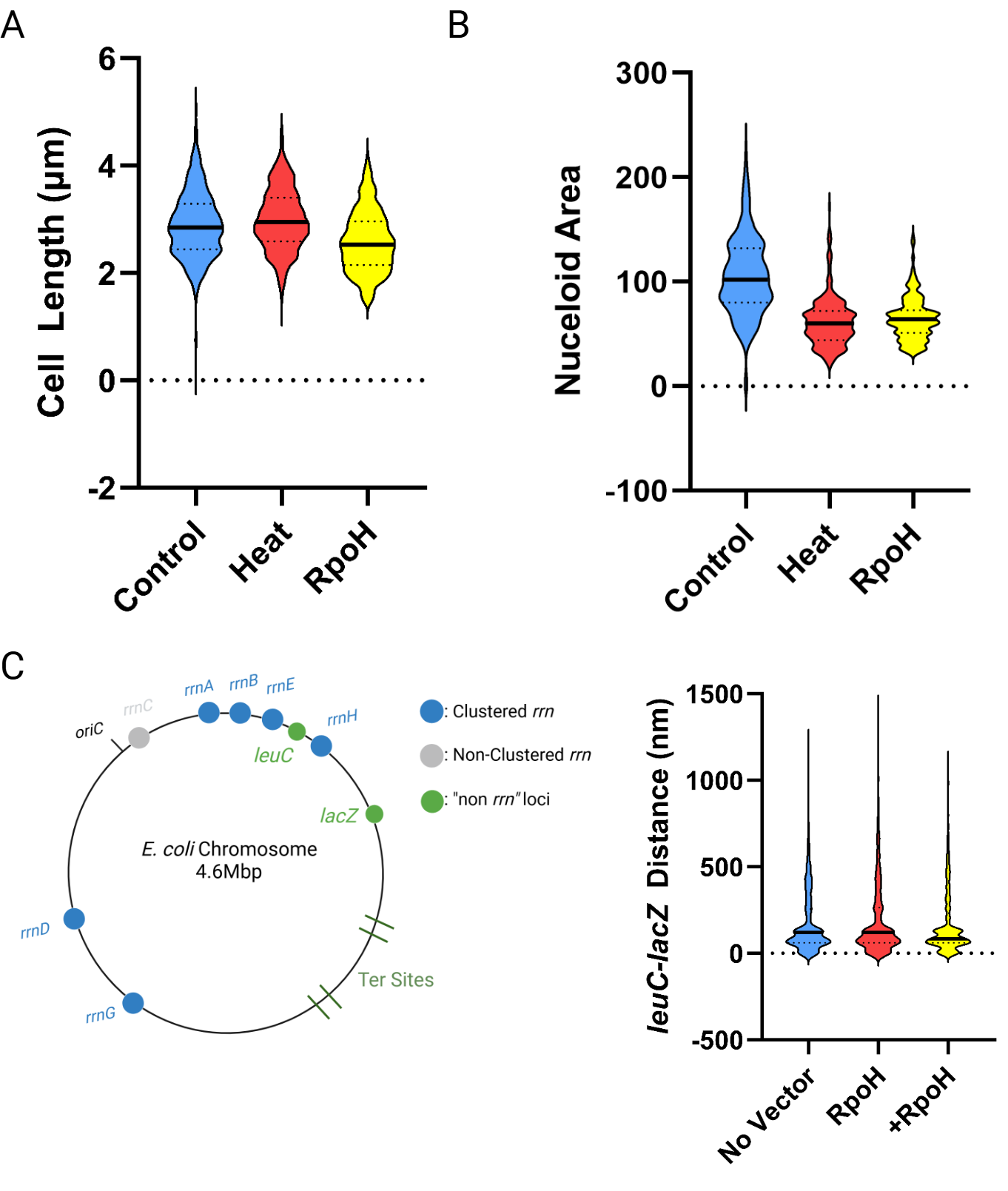

Supplement: S10 Fig — (A) Cell length distribution under heat stress and rpoH overexpression. Conditions for heat stress and rpoH overexpression are identical to that of Figs 1B and 3A, respectively. 200–300 cells were measured for each sample. Data shown are pooled from three biological replicates. (B) Condensation of the nucleoid under the same conditions as in A, with similar cell numbers and replicates. (C) Conditions tested in A do not affect the distance between lacZ and leuC. Left, relative position of lacZ and leuC on the genetic map (not drawn to scale). Right, spatial distance (120 nm, 120 nm, and 85 nm for No Vector, RpoH, and +RpoH, respectively) between lacZ-pMTparS and leuC-P1parS as measured by fluorescent microscopy. Conditions for rpoH overexpression and induction are identical to that of Fig 3A. Data used for plotting S5 Fig graphs can be found in Supplemental “S1 Data” file under appropriate figure headings. Created in BioRender. Ho, K. (2025) https://BioRender.com/a87h018. (TIFF) [file pbio.3003113.s010.tiff]
