# Supplementary material for: Membrane-associated σ factors disrupt rRNA operon clustering in Escherichia coli
Source: PLoS Biol. 2025 Apr 17;23(4):e3003113. doi: 10.1371/journal.pbio.3003113 (PMC12037070; doi:10.1371/journal.pbio.3003113)
Supplement: S1 Table — (DOCX) [file pbio.3003113.s012.docx]

| Strain |  |  |
| --- | --- | --- |
| Strain Name | Genotype or Description | Source |
| MG1655 | Wild-type for all insertion of *parS* seqeuences | Lab Collection |
| HM8305 | Lysogen of Mucts62 | Lab Collection |
| CW45 | Mucts62::CamR lysogen. Cam cassette is inserted at position 35040 | Lab Collection |
| BW25141 | Host for R6K-origin plasmids | Gift of Ian Molineux |
| KH2 | MG1655∆*attB*::FRT | This Work |
| KH3 | MG1655 *yrdA*::P1-*parS*(*rrnD)* *hemG*::pMT-parS (*rrnA)* | This Work |
| KH4 | MG1655 *yrdA*::P1-*parS*(*rrnD)* *clpB*::pMT-parS (*rrnG)* | This Work |
| KH5 | MG1655 *gmhB*::P1-*parS*(*rrnH)* *purH*::pMT-parS (*rrnE)* | This Work |
| KH6 | MG1655 *gmhB*::P1-*parS*(*rrnH)* *murI*::pMT-parS (*rrnB)* | This Work |
| KH7 | MG1655 *gmhB*::P1-*parS*(*rrnH)* *yrdA*::pMT-parS (*rrnD)* | This Work |
| KH8 | MG1655 yeiP::P1-*parS*(*rrnC)* *clpB*::pMT-parS (*rrnG)* | This Work |
| KH9 | MG1655∆*groEL* pKH14 | This Work |
| KH10 | KH2 mlaF::KanR-Mucts62::Cam^r^ | This Work |
| KH11 | MG1655 ∆*nusB*::Kan^r^ | This Work |
| KH12 | MG1655 ∆*rpoZ*::Kan^r^ | This Work |
| KH13 | MG1655 ∆*nc5*::Kan^r^ | This Work |
| KH14 | MG1655 *leuC*::P1-*parS-* Cam^r^ *lacZ*::pMT-parS | This Work |
| Plasmids |  |  |
| Name | Description | Source |
| pKD46 | pSC101 derivative, Source of λred proteins, repA101ts | Lab Collection |
| pKD3 | R6K origin, Cam^r^ | Lab Collection |
| pKD4 | R6K origin, Kan^r^ | Lab Collection |
| pCP20 | Temperative Sensitive Origin, Source of Flp Recombinase | Lab Collection |
| pFHC2973 | pBR322 origin, provide P1 ParB-GFP, pMT-ParB-CFP fusion | Lab Collection [1] |
| pCT3110 | Source of dcas9-3X FLAG | [2] |
| pMAZ-SK CR 2.0 | Source of Cas9 under control of pTet | [3] |
| pMAZ-SK sfgfp | Destination Vector for Golden Gate assembly of gRNA | Gift of Kamyab Javanmardi |
| pBAD33-Flp | Flp Recombinase Under control of pAraBAD | Gift of Chien Hui Ma |
| pSC101 | Low copy vector, Source of pTet-TetA | Lab Collection |
| pKH2 | pBAD33 derivative, pAraBAD-λInt, AmpR | This Work |
| pKH3 | pKD3 derivative, P1-*parS* linked to Cam cassette | This Work |
| pKH4 | pKD4 derivative, pMT-*parS* linked to Kan cassette | This Work |
| pKH5 | Derivative of pCT3110, dcas9-3XFLAG under control of pAraBAD, AmpR | This Work |
| pKH6 | pMAZ-SK sfgfp derivative, gRNA targeting *rrnA* ,KanR | This Work |
| pKH7 | pMAZ-SK sfgfp derivative, gRNA targeting *rrnD*,KanR | This Work |
| pKH8 | pMAZ-SK sfgfp derivative, gRNA targeting *rrnC* ,KanR | This Work |
| pKH9 | pMAZ-SK sfgfp derivative, gRNA targeting *rrnB* ,KanR | This Work |
| pKH10 | pMAZ-SK sfgfp derivative, gRNA targeting *rrnH* ,KanR | This Work |
| pKH11 | pMAZ-SK sfgfp derivative, gRNA targeting *rrnG* ,KanR | This Work |
| pKH12 | pMAZ-SK sfgfp derivative, gRNA targeting *rrnE* ,KanR | This Work |
| pKH13 | pBAD33 derivative, pAraBAD-GroEL/GroES, CamR | This Work |
| pKH14 | pBAD33 derivative, pAraBAD-GroEL, KanR | This Work |
| pKH15 | pBAD33 derivative, pgroE-GroEL, CamR | This Work |
| pKH16 | pBAD33 derivative, pTetA-*rpoH, KanR* | This Work |
| pKH17 | pBAD33 derivative, pTetA-*rpoH* I54N, KanR | This Work |
| pKH18 | pBAD33 derivative, pTetA-*rpoH* L245P, KanR | This Work |
| pKH19 | pSC101 derivative, p*htpG*-*lacZ*, CamR | This Work |
| pKH20 | pBAD33 derivative, pTetA-*rpoH* E265A, KanR | This Work |
| pKH21 | pBAD33 derivative, pTetA-*rpoH* pTetR-*tetR*-*rpoD*, KanR | This Work |
| pKH22 | pKD4 derivative, *attB-sacB* linked to Kan cassette, KanR | This Work |
| pKH23 | pKD4 derivative, attP linked to Kan cassette, KanR | Lab Collection |
| pKH24 | pBAD33 derivative, pTetA-fecI, KanR | This Work |
| pKH25 | pBAD33 derivative, pTetA-*rpoN*, KanR | This Work |
| pKH26 | pBAD33 derivative, pTetA-*rpoS*, KanR | This Work |
| pKH27 | pBAD33 derivative, pTetA-*fliA*, KanR | This Work |
| pKH28 | pBAD33 derivative, pTetA-*rpoE*, KanR | This Work |
| pKH29 | pKH19 derivative, pTetA-*fecI*, pTetR-t*etR*-*rpoD* | This Work |

Table S1. Strains and Plasmids used in this study. Parentheses next to insertion locations indicate the *rrn* operon that is to be labeled.

References

1. Nielsen HJ, Ottesen JR, Youngren B, Austin SJ, Hansen FG. The Escherichia coli chromosome is organized with the left and right chromosome arms in separate cell halves. Mol Microbiol. 2006;62: 331–338. doi:10.1111/j.1365-2958.2006.05346.x

2. Tsui C, Inouye C, Levy M, Lu A, Florens L, Washburn MP, et al. dCas9-targeted locus-specific protein isolation method identifies histone gene regulators. Proc Natl Acad Sci U S A. 2018;115: E2734–E2741. doi:10.1073/pnas.1718844115

3. Ronda C, Pedersen LE, Sommer MOA, Nielsen AT. CRMAGE: CRISPR Optimized MAGE Recombineering. Sci Rep. 2016;6: 19452. doi:10.1038/srep19452
