# Supplementary material for: Membrane-associated σ factors disrupt rRNA operon clustering in Escherichia coli
Source: PLoS Biol. 2025 Apr 17;23(4):e3003113. doi: 10.1371/journal.pbio.3003113 (PMC12037070; doi:10.1371/journal.pbio.3003113)
Supplement: S2 Table — (DOCX) [file pbio.3003113.s013.docx]

| Name | Sequence | Note |
| --- | --- | --- |
| KH882 | CAAGCTTGCATGCAGATTGCAGCATTACACGTTACTCAATTTACTCTTCCTTAAACACAA | Used with 883 to generate P1 ParS insert |
| KH883 | GCAGCTCCAGCCTACACAATCGCTCAAGACCGAAGCCTTAAACTTTCGCCAT |  |
| KH884 | GTCTTGAGCGATTGTGTAGGCTGGA | Used with 885 to generate P1 ParS backbone (pKD3) |
| KH885 | GTGTAATGCTGCAATCTGCATGCAA |  |
| KH888 | CCAGTGCCAAGCTTGCATGCAGATTGCAGCGCTGGTGGGGATAAGGATGC | Used with 889 to generate pMT ParS insert |
| KH889 | CAGCCTACACAATCGCTCAAGACGTGTAATGCGAAATTATGAGTCACGAAGAGGT |  |
| KH890 | ATTACACGTCTTGAGCGATTGTGT | Used with 889 to generate pMT ParS backbone (pKD4) |
| KH891 | GCTGCAATCTGCATGCAAGCT |  |
| KH955 | GGAGCACGCACCAATATCCAGGATGGCAGTTTGCATGCAGATTGCAGCATTAC | Used with 956 to insert P1-parS at *yrdA* (rrnD) |
| KH956 | GGGTTGTACGAGGATTTATGAGTGACATGCAACATGCCATGGTCCATATGAATATCCTCC |  |
| KH1140 | ATGTACATTATGTACAGATCGGAGCACGCACCAATATCCTTGCATGCAGATTGCAGCAGG | Used with 1141 to insert pMT-parS at *yrdA* (rrnD) |
| KH1141 | GGATTTATGAGTGACATGCAACATACTGCCATCCTGCCATGGTCCATATGAATATCCTCC |  |
| KH961 | GCGCGAGATTGCCTCCTACCTGGCTTCGGAACTGAAATTGCATGCAGATTGCAGCATTAC | Used with 962 to insert P1-parS at hemG (rrnA) |
| KH962 | AGTTTTCCCACTGTGGTTCTTCAATGCGGTGCACAGCCATGGTCCATATGAATATCCTCC |  |
| KH927 | CCATACTGCGCTTCTTTTTATCGCTCATTCAATTGCATGCAGATTGCAGCAGG | Used with 928 to insert pMT-parS at *yieP* (rrnC) |
| KH928 | CTGCTACGCTCACCTGATAAGTGATAACCCGATAAGCCATGGTCCATATGAATATCCTCC |  |
| KH1220 | TGGATGGGCGGTGAAATATCCCTGCGTAGTGCAGAAAAATTGCATGCAGATTGCAGCAGG | Used with 1221 to insert pMT-parS at *murI* (rrnB) |
| KH1221 | CTAATCATGCGGTAGAAGAGGTCAGACTACGCAAAGCCATGGTCCATATGAATATCCTCC |  |
| KH1135 | AACGGGCAACGCCGTCAATGAAATGGATGTTAAGGTTGCATGCAGATTGCAGCATTACAC | Used with 1136 to insert p1-parS at *gmhB* (rrnH) |
| KH1136 | TTCGCATAAACCTGATTGATTTAGACGTCTGGATGGCCATGGTCCATATGAATATCCTCC |  |
| KH1100 | GGCTAGCAGTTGTCCGCCGATCAACGTCACATACTGAAATTGCATGCAGATTGCAGCAGG | Used with 1101 to insert p1-parS at *clpB* (rrnG) |
| KH1101 | TTGCCGTTGAAGGGCGCAAAGGTTTTTACGCATCAGCCATGGTCCATATGAATATCCTCC |  |
| KH1132 | CATCTCTTTAATAATGGCGTCATAGTCGCTGCTCTTCACTTGCATGCAGATTGCAGCAGG | Used with 1133 to insert pMT-parS at *purH* (rrnE) |
| KH1133 | CCGCCGCCAAGAACCATAAAGATGTCGCAATCGTGGCCATGGTCCATATGAATATCCTCC |  |
| KH454 | GAGCGGATAACAATTCCCCTCTAGA | Used with 455 to generate backbone for pKH2. Template is pCT310 |
| KH455 | TCATCGGTATCATTACCCCCATG |  |
| KH456 | TTTCTGTTCATGGGGGTAATGATACCGATGACATCGATGCATAATGTGCCTGTC | Used with 455 to generate pAraBAD insert for pKH2. Template is pBAD33-Flp |
| KH457 | TTATTTCTAGAGGGGAATTGTTATCCGCTCCGGGTATGGAGAAACAGTAGAGAG |  |
| KH440 | TAGTTCTCAGCCAGAACATACGAA | Used with KH441 to make gRNA insert targeting *yeiP* (rrnC) |
| KH441 | AAACTTCGTATGTTCTGGCTGAGA |  |
| KH442 | TAGTCGCGATCTTTTTCCACAAAT | Used with KH443 to make gRNA insert targeting *yrdA* (rrnD) |
| KH443 | AAACATTTGTGGAAAAAGATCGCG |  |
| KH480 | TAGTCTATACCGATTGGGAGCAGG | Used with KH481 to make gRNA insert targeting *hemG* (rrnA) |
| KH481 | AAACCCTGCTCCCAATCGGTATAG |  |
| KH482 | TAGTCTCGAAAAACTGGCAGTTTT | Used with KH483 to make gRNA insert targeting *murI* (rrnB) |
| KH483 | AAACAAAACTGCCAGTTTTTCGAG |  |
| KH484 | TAGTCTGAGCAGAGCGCGGCGGAC | Used with KH485 to make gRNA insert targeting *purH* (rrnE) |
| KH485 | AAACGTCCGCCGCGCTCTGCTCAG |  |
| KH486 | TAGTGATAAAAAAGCAGCAAAAAC | Used with KH487 to make gRNA insert targeting *gmhB* (rrn*H*) |
| KH487 | AAACGTTTTTGCTGCTTTTTTATC |  |
| KH488 | TAGTTCCGGTCTTCATTAACTTCC | Used with KH489 to make gRNA insert targeting *clpB* (rrnG) |
| KH489 | AAACGGAAGTTAATGAAGACCGGA |  |
| KH451 | TAGTGTCACGACGTTGTAAAACGA | Used with KH452 to make gRNA insert targeting *lacZ* |
| KH452 | AAACTCGTTTTACAACGTCGTGAC |  |
| KH337 | TAGTCGTTTTACAACGTCGTGACT | Used with KH481 to make gRNA insert targeting *lacZ* (no PAM) |
| KH338 | AAACAGTCACGACGTTGTAAAACG |  |
| KH965 | GTTTTTTTGGGCTAGCGAATTCGAGCTCGGCGCACGACACTGAACATACGAATTT | Used with KH966 to generate groEL insert |
| KH966 | TGCAGGTCGACTCTAGAGGATCCCCGGGTAGAGGTGCAGGGCAATTACATCAT |  |
| KH967 | TACCCGGGGATCCTCTAGAGTC | Used with KH968 to generate backbone for KH13. Template is pBAD33-Flp |
| KH968 | CCGAGCTCGAATTCGCTAGCC |  |
| KH1012 | AAAGCGACATTCTGGCAATTGTTGAAGCGTAATCCGATTACACGTCTTGAGCGATTGTGT | Used with KH1013 to generate PCR product for deletion of *groL* |
| KH1013 | CAGACATTTCTGCCCGGGGGTTTGTTTATTTCTGCGCCATGGTCCATATGAATATCCTCC |  |
| KH1035 | CGTTTTTTTGGGCTAGCGAATTCGAGCTCGGTCACCCATAACAGATACGGACTTTC | Used with KH1036 to genrate insert for pKH13 |
| KH1036 | CTGTTTTATCAGACCGCTTCTGCGTTCTGATCAAAAGAAAAACCCCCAGACATTTC |  |
| KH1164 | ATCAGAACGCAGAAGCGGTCTG | Used with KH1165 to genrate backbone for pKH14 |
| KH1165 | ATAGGGTTTGCAGAATCCCTGCTTC |  |
| KH1166 | TGGACGAAGCAGGGATTCTGCAAACCCTATTGTCCGATTGCGCCCAAATTTTGG | Used with KH1167 to generate insert for pGroE-GroEL/GroES |
| KH1167 | TGTTTTATCAGACCGCTTCTGCGTTCTGATGGCAATTACATCATGCCGCCC |  |
| KH1214 | ATCAGAACGCAGAAGCGGTCTG | Used with KH1215 to generate insert for KH15 |
| KH1215 | GAGAAAGTCCGTATCTGTTATGGGT |  |
| KH1216 | GCGTCACCCATAACAGATACGGACTTTCTCGCCAGAAGATATCGATTGAGAGGATT | Used with KH1217 to generate insert for KH15 |
| KH1217 | TTAAATTCGTATGTTCAGTGTCGTGCGCGGTCCAGGGTTCTCTGCTTAATAGC |  |
| KH1308 | ACTTTGCTTCATATGACGAAGCAACC | Used with KH1309 to generate insert for pKH16 |
| KH1309 | GCGCCTATATCGCCGACATCAC |  |
| KH1310 | GATGTCGGCGATATAGGCGCTTAATCGTCCAGGAAGCTACG | Used with KH1309 to generate backbone for pKH16 |
| KH1311 | TTCGTCATATGAAGCAAAGTAAGGACATCGGGTCAATCGCC |  |
| KH1277 | CAGCTGGAAAAGAACGCGATGAAAAA | Used with KH1278 to make pKH17 |
| KH1278 | GCGTACACGCGCAGCGGAAA |  |
| KH1285 | GCGCTGGCCGGACGAAGA | Used with KH1286 to make pKH18 |
| KH1286 | GCACGGATGATGTCCTGGCT |  |
| KH1308 | TAAAACGCTGAACCTGTCTCACCTG | Used with KH1268 to make pKH19 backbone |
| KH1309 | GCTGCTTCCAGATCGCCATG |  |
| KH1310 | GATGTCGGCGATATAGGCGCTTAATCGTCCAGGAAGCTACG | Used with KH1311 to make pKH19 insert |
| KH1311 | TTCGTCATATGAAGCAAAGTAAGGACATCGGGTCAATCGCC |  |
| KH2215 | CTATTAAGCAGAGAACCCTGGATGAGA | Used with KH2216 to make backbone for pKH22-pKH27 |
| KH2216 | TCAAATCCTCTCAATCGATATCTTCTGGC |  |
| KH2217 | TGCCAGAAGATATCGATTGAGAGGATTTGAATGTCTGACCGCGCCACTAC | Used with KH2219 to make insert for pKH22, and insert for pKH27 |
| KH2218 | GATTCTCATCCAGGGTTCTCTGCTTAATAGTCATAACCCATACTCCAGACGGAA |  |
| KH2219 | TGCCAGAAGATATCGATTGAGAGGATTTGAATGAGTCAGAATACGCTGAAAGTTCATG | Used with KH2221 to make insert for pKH23 |
| KH2220 | GTTCTGATTCTCATCCAGGGTTCTCTGCTTAATAGTTACTCGCGGAACAGCGCTT |  |
| KH2221 | TGCCAGAAGATATCGATTGAGAGGATTTGAATGAGCGAGCAGTTAACGGACC | Used with KH2223 to make insert for pKH24 |
| KH2222 | GATTCTCATCCAGGGTTCTCTGCTTAATAGTCAACGCCTGATAAGCGGTTGAAC |  |
| KH2223 | TGCCAGAAGATATCGATTGAGAGGATTTGAATGAAGCAAGGTTTGCAACTCAGG | Used with KH2225 to make insert for pKH25 |
| KH2224 | GATTCTCATCCAGGGTTCTCTGCTTAATAGTCAAACGAGTTGTTTACGCTGGTTTG |  |
| KH2225 | TGCCAGAAGATATCGATTGAGAGGATTTGAGTGAATTCACTCTATACCGCTGAAGG | Used with KH2227 to make insert for pKH26 |
| KH2226 | GATTCTCATCCAGGGTTCTCTGCTTAATAGTTATAACTTACCCAGTTTAGTGCGTAACC |  |
| KH1262 | TTTTTTTGTCCGCCAGGATCCAGGCGTGATCCGCAATACTTAACACTTCCTCCTAATTTTTGTTGACACTCTATC | Used with KH1263 to generate PCR product to insert *attP* into *mlaF* |
| KH1263 | TTTCCCTTTGATGGCACATCGTGAGAAACCACCACACAAGTCACGCCCAGCGCGCTGTTCAGGATGGTCGGGTTTAACGTTCATT |  |
| KH1717 | TTTTTGCGGCCGCAAGATCCG | Used with KH1672 to make backbone for KH20 |
| KH1672 | TGATGTTATTCCGCGAAATATAATGACCCT |  |
| KH1716 | GTTGAACTGCGGATCTTGCGGCCGCAAAAATGATGTTATTCCGCGAAATATAATGACCCT | Used with KH1675 to make insert for KH20 |
| KH1675 | AGGGTCATTATATTTCGCGGAATAACATCAGATGGTCGGGTTTAACGTTCATT |  |
| KH1447 | CCATCGGTGATGTCGGCGATATAGGCGCATTTCACCGTCATCACCGAAACGC | Use with 1448 to make insert for KH19 |
| KH1448 | TTCAAATCCTCTCAATCGATATCTTCTGGCCGAGCTCGAATTCCATGGTCTGTTTC |  |
| KH1449 | GCCAGAAGATATCGATTGAGAGGATTTGA | Use with 1448 to make backbone for KH19 |
| KH2506 | CAACTGGTAATGGTAGCGACCGGCGCTCAGCTGGAATTCGCCAAGCTTGCATGCAGATTG | Use with KH2507 to insert P1-*parS* at *leuC* |
| KH2507 | TTCCCAACAGTTGCGCAGCCTGAATGGCGAATGGCGCCATGGTCCATATGAATATCCTCC |  |
| KH2508 | TTGCGAATGTCGGCGAAATGTCCGGTCACAGCAGCACTTGCATGCAGATTGCAGCATTAC | Use with KH2509 to insert pMT-*parS* at *lacZ* |
| KH2509 | CATGGCTAAGACGTTATACGAAAAATTGTTCGACGGCCATGGTCCATATGAATATCCTCC |  |

Table S2. Primers Used in this study.
