# Supplementary material for: Membrane-associated σ factors disrupt rRNA operon clustering in Escherichia coli
Source: PLoS Biol. 2025 Apr 17;23(4):e3003113. doi: 10.1371/journal.pbio.3003113 (PMC12037070; doi:10.1371/journal.pbio.3003113)
Supplement: Attachment — Submitted filename: Response to Reviewers.docx [file pbio.3003113.s024.docx]

Reviewer #1: In a previous study, the group of Prof. Harshey provided evidence for long-range interactions on the E. coli chromosome. Using a Mu-based transposition assay, they found that rrn loci clustered together, despite being located across the E. coli chromosome. They identified some DNA elements in the region of these loci as being essential for the clustering. In the present study, Ho and Harshey probe for proteins that may mediate this clustering. For this, they first screen for stress conditions where the clustering is disrupted. They identify ethanol and heat-shock stresses as two conditions where clustering is reduced. Next, they carry out a pull down (via dCasd9 targeting) of the clustered rrn operon regions. They find that GroEL is common across 4 of the rrn operons. Further probing reveals that perturbation in the GroEL-GroES ratios affects clustering, and this may be driven by sigma factor competition. The authors go on to shown that sigmaH (expressed during heat shock) can disrupt clustering, likely via competition with sigmaD. Mutational analysis suggests that this is not dependent on active transcription but relies on sigmaH association with DNA and its RNA polymerase interactions.
The study is interesting as it probes into the mechanism of rrn operon clustering in E. coli. Given the limited examples of such clustering in bacterial genomes, the findings will be of broad relevance to the chromosome biology community across bacteria and eukaryotic systems. I do have some points that authors should consider.

>We thank the reviewer for suggesting productive experiments that have provided unexpected and interesting results detailed below.

1.      Comparison across diverse stressors. Authors use a diverse variety of stressors to assess which conditions affect rrn clustering. However, comparison across these conditions is challenging as they may not affect cell growth to the same extent. It is unclear what concentrations/ range of stress conditions were tried to enable reliable comparison across stringent response, cold shock, ethanol and heat shock.

> We are not quite sure exactly what the reviewer means about ‘reliable comparison’ across the multiple stressors, given that they produce different responses. We chose stressors that are well-defined in the literature and tried to use those exact conditions where possible with the expectation that if any one of them could perturb *rrn* clustering, we might be able to determine the causative agent. For a majority of these stressors, there are no specific phenotypes, and the stress is manifested as changes in RNA and protein profiles, the exception being SHX, for which we confirmed that addition of SHX caused a cessation of growth at the time of imaging. For ethanol, we employed a lower dose (0.5% instead of the typical >2.5%) due to initial concerns that membrane stress might result in a false-positive de-clustering of *rrn*. Since we observed a dramatic change in heat stress, we did not attempt to further validate the other stress conditions.

2.      Microscopy observations:
a.      In all stress conditions, cell lengths and global chromosome condensation/ organization is subject to change when compared to wildtype. Hence it is insufficient to measure inter-locus distance between rrn loci alone. Authors should provide cell length distributions and DAPI staining analysis in all imaged conditions to show that cell lengths and chromosome compaction are comparable to wild type, even though the inter-locus distance is changing.

> This suggestion provided interesting unanticipated results provided in Fig. S8. We examined cell length distributions and nucleoid area for cells under heat stress or *rpoH* overexpression (Fig. S8A and B, respectively), and found that cell length under the former changed minimally (Fig. S8A), but that under the latter, cells were shorter on average (2.5µm vs 2.8µm of Wt). Nucleoids appeared to be more compact under both conditions (Fig. 8B). Though we do not have an explanation for these observations, we interpret the results to mean that the de-clustering effect of these are not a consequence of changes in cell length or nucleoid decompaction. A description of these experiments is on lines 230-242.

b.      In addition, authors should measure the inter-locus distance between two chromosome loci known to not cluster, as a control to show that the sigma factor effects are specific to the rrn operons.

> In response to this comment we examined two non-*rrn* loci (*leuC* and *lacZ*) specifically, since they lie in a genomic region that is not known to interact with *rrn* (at least *rrnD*) described previously (Walker et al. 2020) (Fig. S8C, left). We found that *rpoH* overexpression does not affect localization of these two loci (Fig. S8C, right).

c.      It is unclear what microscope was used for the imaging (details are missing in the methods). If this is widefield imaging, do the authors have the resolution power to differentiate across 216-230nm? In my experience any localizations below 250nm distances are not resolvable by widefield microscopy techniques and hence commenting on distance variations as small as a change from 216nm to 230nm is not reliable.

> We have added the microscope model and camera details to the methods section (Line 551-552). We apologize for this oversight. As rightly pointed out, distances below 250nm are not possible to resolve, especially since we are not using sub-pixel algorithm to analyze the data. We have now called attention to this in the text (lines 97-99) where the situation is first encountered (Fig. 2) and added an explanation to Fig. 2 legend as well. We had determined an arbitrary cutoff point so as not to introduce confusion regarding which data points we considered worth following up on. In other words, unless s shift of more than 2.5 fold (over the control) was evident, we considered them the same.

d.      The violin plots of distances between loci have values below zero as well - is this possible?

> The data set has no points below zero. This is an unintended consequence of GraphPad trying to draw a border to cover through all the data points. Since we have some data points that are “0” in value, the program tries to draw the border of the graph to encompass these points, which results in the border going below zero. We have added an explanation for this point in Fig. 2 legend. We apologize for the confusion.

3.      dCas9 pull-down experiment: This is an excellent experiment for discovering proteins bound to these clusters. However, since this requires formaldehyde crosslinking, do the authors have confidence that any cluster-specific protein will still remain bound and be pulled-down? Authors need to clearly discuss this caveat of the experimental design.

> We have added this potential pitfall to the main text (Lines 173-178)

4.      Role of sigmaH in rrn operon clustering. Given that there could be global transcriptional changing during heat shock/ sigmaH overexpression, the decrease in rrn clustering could be due to more global changes to chromosome organization. Have the authors conducted experiments to see whether the sigmH-dependent declustering is specific to rrn operons, or whether such over-expression triggers more global changes in chromosome organization/ transcription (voa RNA-seq/ chromosome capture methods)?

> While we observed that the overexpression of σ^H^ causes global changes to the nucleoid (Fig. S8B) and to global chromosomal contacts as reported by Mu transposition (Fig. 6D), we also note that the de-clustering activity of σ^H^ is independent of its transcriptional activity (Fig. 4C, I54N mutant), thus we do not think that RNA-seq would reveal the agent responsible for clustering of *rrn*. We did not attempt the more traditional Hi-C approach since *rrn* clustering has not been observed by this method.

5.      Discussion section: This section is rather long, and mostly recapitulates the results. Authors should tighten this section significantly. The model suggests that sigmaH binds the inner-membrane and causes declustering. However, I am not clear as to how the inner membrane binding would cause this declustering. The model figure does not provide much insight into this proposed mechanism.

>We have shortened the Discussion by 14 lines and modified the model in Fig. 7 for clarity. As we have suggested, binding of σ^H^ at the inner membrane would be capable of dislodging membrane bound σ^D^ (supported by rescue of σ^D^ co-expression with either σ^H^ or FecI, Fig. 4A and Fig. 5D), which, we suspect, is responsible for clustering.

Minor comments:
1.      Scale bar is missing in microscopy images

> We apologize for this oversight. Scale bars have been added.

2.      Please provide details of the number of biological replicates in each of the violin plots. Were these data pooled across replicates?

>The data shown is for one of three biological replicates. We typically measure 300-800 foci per replicate, but usually around 700. Pooling them would create an unusually large number of foci, at least in comparison to the literature that we are familiar with, so we did not pool them. We have added this to Fig. 2 legend.

3.      Upset plot in Fig. 3A is not readable

>We have moved the Upset plot to its own Figure (Fig. S5) to improve readability.

4.      Please check the grammar for the discussion section L254-258.
> “of rRNA operons” was missing from the text, now fixed (L348-349).

Reviewer #2: The E.coli chromosome has seven ribosomal RNA operons and six of them show clustered spatial localization in the cell. In this manuscript by Ho and Harshey, the authors perturbed the cell using physical and chemical stresses including amino acid starvation, heatshock, coldshock and ethanol shock, and found that ethanol shock and heatshock disrupted the clustering. The authors went on the show that it is the elevated level of the sigma factor RpoH that disrupts the clustering of rrn operons. A similar effect was observed when FecI was expressed. Both RpoH and FecI associate with the cell membrane and both were counteracted by expression of sigma factor RpoD. The authors concluded rrn operons are clustered at the cell membrane directly or indirectly by RpoD.

This is a solid study involving a complementary approaches. The experiments were carefully executed, the results were critically analyzed and interpreted. The topic will be of broad interest to researchers that study chromosome biology field. However, I found some major gaps of understanding (see below). Filling these gaps could strengthen the authors' model:

> We thank the reviewer for highlighting both conceptual and technical gaps in the experiments in the manuscript. We have addressed some essential concerns below.

1)      One important component of the model is rrns are clustered at the cell membrane. The authors have a lot of images for the localization of rrns in the cells. When analyzing these data, is there evidence that the rrn loci are associated with the cell membrane? In other words, is there a tendency for the foci to be at the periphery of the cell?

>This is a question that we are actively pursuing using super-resolution microscopy. In the images shown in this manuscript, we have not observed a marked tendency for *rrn* loci to localize to the periphery. However, this does not preclude them from being localized to the membrane since we do not have Z-axis information.

2)      Figure 2D. Why does heatshock reduce the copy number of rrnA but not rrnD? Because the number of foci are different, the absence of colocalization might be overestimated. In Figure 2CD or an SI figure, the authors should show a different pair of loci that have similar number of foci before and after RpoH expression.

>We are unable to explain why *rrnA* is reduced to one focus upon heat shock. However, *rpoH* overexpression did not show a similar reduction in *rrnA* foci as now stated on lines 213-214 and the data shown in Fig. S7.

3)      GroEL is pulled down from 4/7 rrn operons. For dCas9 pulldown. It is unclear whether GroEL specifically get enriched at the rrn operons or it just has higher levels upon stress and get enriched everywhere. To distinguish these two possibilities, I suggest the authors do repeat dCas9 pulldown using one or two control loci that are not close to any rrn and examine GroEL enrichment.

> We had indeed considered that general protein abundance and/or DNA binding activity might interfere with what we would consider a “hit”. We had therefore employed gRNA targeting *lacZ*, which resides in a genomic area that is not known to interact with at least *rrnD* (Walker et al. 2020) as a pulldown control. Targets identified in gRNA targeting *rrn* were then normalized to the *lacZ* gRNA control, which should account for abundance. This had been mentioned in Methods, but we have now added it to the text on lines 173-178.

4)      Figure 6: Mu experiment. It looks like the strongest effect RpoH has is on the overall landscape of genome folding, and not specifically affecting rrn clustering. Then the question is: does RpoH specifically disrupt rrn flustering or does it lower chromosome interactions overall and the declustering of rrns is just a consequence of chromosome reorganization? Figure 6E-J showed lower interactions between rrnD and other rrns upon RpoH expression, does rrnD have lower interactions with non-rrn sites as well? If the authors have a Mu inserted in a non-rrn region (say geneX), then look at its interactions with regions (gene y, z, w, t etc) that are the same distance away as rrns away from rrnD, then measure the interaction between x-y, x-z, x-w, x-t, etc, before and after RpoH expression, does RpoH lower the interaction of these other loci pairs as well?

>We have not looked at Mu transposition from a non-*rrn* locus in the RpoH background. However, as stated in the response to Reviewer 1, we have now examined the effect of *rpoH* overexpression on the nucleoid and on two non*-rrn* loci. We found that both heat stress and *rpoH* overexpression reduce the nucleoid area (Fig. S8B). This is consistent with the result from Mu transposition (Fig. 6B and C), which suggested that local contacts are favored during *rpoH* overexpression. We also examined two loci that should not interact with *rrn* (*leuC* and *lacZ,* Fig. S8C, Left). We observed that these two loci do not exhibit the same de-clustering upon *rpoH* overexpression (Fig. S8 C, Right). Also, we can restore clustering to the RpoH-elevated strain with RpoD or FecI (Fig. 5D), indicating that the de-clustering effect of *rpoH* on *rrn* operons cannot simply be attributed to its global effect on the nucleoid alone.

5)      Figure 7 model: Where should be GroEL put in this model? RpoD not only bind to rrn promoters, but also bind to promoters of many housekeeping genes. What is preventing these other genes getting incorporated into the rrn clusters? Does the model predict that other highly transcribe genes (outside of rrn operons) are clustered with the rrns? Does Mu result support the idea the higher the expression of a gene, the more interactions it has with a rrn?
>We surmise that the repression of σ^H^ activity by GroEL is the chief reason we observed a de-clustering effect in strains with artificially controlled GroEL. Therefore, under clustering conditions, GroEL prevents σ^H^ from replacing σ^D^ at the inner membrane. Upon membrane stress, unfolded substrates relieve the inhibition of σ^H^ by GroEL by occupying GroEL for re-folding, thus allowing de-clustering to occur. The confounding factor is that transcription does not account for clustering since (1) neither SHX nor Rif result in de-clustering, and (2) *rrnC*, which as far as we are aware, is as proficient for transcription as other any other *rrn* loci, does not cluster. Therefore, productive transcription is not the issue. To the best of our knowledge, the *rrn* operons are the highest transcribed genes (80% of transcripts are *rrn*) and no other genes come close. We have provided a modified Fig. 7 to include GroEL.


Reviewer #3: Review of Ho and Harshey, clustering of rRNA operons in E. coli is disrupted by sigma H This manuscript is a follow up of the Harshey lab's seminal paper published in Cell in 2020, Walker et al. 180: 703-716. In that work, the authors found that 6 of the 7 rRNA operons in an E. coli cell (all except rrnC) are clustered in space in vivo. The authors find, using the Phage Mu method employed in the Cell paper and also by fluorescence microscopy using tags on the rRNA operons on the chromosome, that this clustering phenomenon is disrupted by heat shock or by expression of sigma 32 or sigma FecI, two inner membrane-associated sigma factors, but not by other sigma factors. Even a small increase in sigma 32 levels is sufficient for de-clustering, but transcription by Esigma32 is not required. Co-expression of sigma 70 with sigma 32 is sufficient to rescue clustering, suggesting that sigma 32 competes with sigma 70 to cause de-clustering of the physical contacts between the rRNA promoter regions. In an attempt to use mass-spec to identify proteins that cause clustering, the authors identify GroEL as making a contribution to clustering of 4 of the 6 clustered operons. The authors address other possible contributors, including condensate formation, concluding that RNAP condensates are a feature of highly active transcription units, but since neither NusB or RpoZ impact clustering whereas they are crucial for condensate formation, condensates are not responsible for rRNA operon clustering. In general, I found this paper very clear and well-written. I have only very very minor comments.

>We thank the reviewer for their careful reading of the manuscript and for correcting several errors and omissions. We have made all the suggested edits.

1.      In many figures, fluorescent images are superimposed on phase contrast images. However, these are referred to as "bright field". My understanding is that these images are phase contrast, not "bright field" (which does not mean the same thing as phase contrast). Please correct the text, legends, and methods.

>Corrected.

2.      I had some difficulty determining on line 136 which panel in Fig. 3 the authors are referring to. I think it's the "red" plot that shows the increased distance, but describing this as the third plot is a bit confusing.

>We now say ‘third plot, red, from left’, which should clarify (line 185).

3.      On line 139 the authors refer to Fig. 2D. Should this be Fig. 3D?

>Yes. This is now fixed.

4.      Would it be useful to point out in the text that the numbers above the plots in Figures 2B, 3D and E, and 4A and C are the numbers of pairs monitored, not the average distances between the rRNA operons? I found this confusing at first. An alternative solution would be to move the number of measured foci pairs to the legend or Methods (just saying 300-800 would be sufficient) and including the actual distances measured on the figure, so they match the text.

> We have removed the number of foci observed and opted to introduce the median distance in the graph. The number of foci observed is now given as a range for the data points concerned in the legends.

5.      Line 357. The order of RNAP subunit assembly is alpha2 ◊ alpha2- beta ◊alpha2- beta- beta prime ◊ alpha2- beta- beta prime- omega. The earlier referenced Ishihama pathway was before recognition of the importance of omega for assembly and was superseded by the later reference (ref. 51). Including the earlier pathway might be confusing.

>We have removed the former pathway (line 440).

6.      Fig 1. Perhaps it should be mentioned in the legend that the positions of the operons on the circular chromosome are not to scale.

>This detail is added.

7.      The generic E. coli rRNA operon shown in Fig. 1 is pictured without a distal tRNA and is labeled as having a "variable tRNA" in the 16S-23S spacer. Three E. coli rRNA operons have a tRNA or two distal to the 23S or 5S genes. Perhaps to be more precise, it might be best to label the schematic operon as rrnG and indicate the identity of the spacer tRNA as tRNAGlu2 and also say that some operons have distal tRNAs.

>Fig. 1 and its legend are amended as suggested.

8.      I found a couple of typos: In ref. 37, Ciaran is the author's first name, the last name is Condon. Also, on line 400, the text should be "use of non-Mu" …

>These issues have been fixed.
